# Automated mechanical peripheral stimulation for gait rehabilitation in Parkinson's disease: A comprehensive review

**DOI:** 10.1016/j.prdoa.2023.100219

**Published:** 2023-09-23

**Authors:** Roberto Tedeschi

**Affiliations:** Department of Biomedical and Neuromotor Sciences, Alma Mater Studiorum, University of Bologna, Bologna, Italy

**Keywords:** Parkinson's disease, Automated mechanical peripheral stimulation (AMPS), Freezing of gait, Motor performance, Physical therapy

## Abstract

**Background:**

Automated Mechanical Peripheral Stimulation (AMPS) has emerged as a potential rehabilitative intervention for gait abnormalities in Parkinson's disease (PD). However, the long-term effects and combined therapy with physical exercise remain unclear. This review aimed to explore the effects of automated mechanical peripheral stimulation (AMPS) on gait and motor performance in individuals with Parkinson's disease (PD).

**Methods:**

A research was conducted in relevant databases to identify studies investigating the effects of AMPS on gait and motor outcomes in PD patients. Inclusion criteria were set based on Population, Concept, and Context (PCC) criteria. Data extraction and analysis were performed to synthesize the findings.

**Results:**

Ten studies met the inclusion criteria and were included in the review. The studies collectively demonstrated positive effects of AMPS on gait parameters, such as walking velocity, stride length, and walking stability. Some studies also reported improvements in functional performance and muscle activation during walking.

**Conclusions:**

The findings suggest that AMPS holds promise as a potential intervention to improve gait and motor performance in individuals with PD. However, the evidence is limited, and further well-designed randomized controlled trials are needed to establish the long-term efficacy and optimal protocols for AMPS in PD rehabilitation.

## 1 Introduction

Parkinson's disease (PD) is a complex neurodegenerative disorder characterized by the progressive degeneration of dopaminergic neurons in the substantia nigra, leading to a wide range of motor and non-motor symptoms [1], [2]. Among the motor symptoms, gait abnormalities and freezing of gait (FOG) [3] are particularly debilitating, significantly impacting mobility and overall quality of life in PD patients [4]. Non-pharmacological interventions have gained increasing attention as complementary approaches to manage gait disturbances in PD [5], [6]. One such intervention is Automated Mechanical Peripheral Stimulation (AMPS) [7], a novel rehabilitative technique that targets both peripheral and central sensitivity disturbances observed in PD patients. AMPS delivers mechanical pressure stimulations to specific areas of the feet, aiming to correct gait abnormalities and enhance motor performance [8]. While previous studies have shown promising outcomes with AMPS interventions, there remains a critical gap in our understanding of its underlying effects on gait biomechanics [3], [7], [9], [10], [11]. Comprehensive investigations exploring the long-term impact of AMPS on gait parameters, muscle activation patterns, and functional outcomes in PD patients are scarce in the scientific literature. Therefore, the purpose of this review is to rigorously analyze the existing scientific literature on AMPS interventions in individuals with Parkinson's disease, with a primary focus on both functional performance and gait biomechanics. By synthesizing and critically evaluating the findings from relevant studies, this review aims to provide a scientifically robust assessment of the efficacy and potential mechanisms of AMPS in improving gait disturbances in PD [12]. Furthermore, we intend to identify research gaps and methodological limitations in the current body of literature, as well as propose avenues for further investigation. A deeper scientific understanding of the effects of AMPS on gait biomechanics and functional outcomes can offer valuable insights to clinicians, researchers, and rehabilitation specialists, guiding evidence-based treatment strategies for optimizing gait rehabilitation in Parkinson's disease [13], [14]. Through this review, we seek to contribute to the advancement of knowledge in PD rehabilitation, ultimately paving the way for evidence-based and personalized interventions that can improve the overall well-being and mobility of individuals living with PD and gait impairments (see Table 1)..

**Table 1:** Main characteristics of included studies.

N°	Author	Title	Year	Country	Study Design	Source of evidence	Level of performance
1	Kleiner A et al. [7]	The Parkinsonian Gait Spatiotemporal Parameters Quantified by a Single Inertial Sensor before and after Automated Mechanical Peripheral Stimulation Treatment	2015	Italy	Trial	Traditional	Not reported
2	Stocchi F et al. [17]	Long-term effects of automated mechanical peripheral stimulation on gait patterns of patients with Parkinson's disease	2015	Italy	Trial	Traditional	Not reported
3	Pagnussat AS et al. [18]	Plantar stimulation in parkinsonians: From biomarkers to mobility - randomized-controlled trial	2018	Brasil	Trial	Traditional	Not reported
4	Galli M et al. [10]	Peripheral neurostimulation breaks the shuffling steps patterns in Parkinsonian gait: a double blind randomized longitudinal study with automated mechanical peripheral stimulation	2018	Italy	Trial	Traditional	Not reported
5	Kleiner AFR et al. [3]	Automated Mechanical Peripheral Stimulation Effects on Gait Variability in Individuals With Parkinson Disease and Freezing of Gait: A Double-Blind, Randomized Controlled Trial	2018	Italy	Trial	Traditional	Not reported
6	Prusch JS et al. [19]	Automated mechanical peripheral stimulation and postural control in subjects with Parkinson's disease and freezing of gait: a randomized controlled trial	2018	Brasil	Trial	Traditional	Not reported
7	Pinto C et al. [20]	Automated Mechanical Peripheral Stimulation Improves Gait Parameters in Subjects With Parkinson Disease and Freezing of Gait: A Randomized Clinical Trial	2018	Brasil	Trial	Traditional	Not reported
8	Pagnussat AS et al. [11]	Plantar stimulation alters brain connectivity in idiopathic Parkinson's disease	2020	Brasil	Trial	Traditional	Not reported
9	Zelada-Astudillo N et al. [12]	Effect of the combination of automated peripheral mechanical stimulation and physical exercise on aerobic functional capacity and cardiac autonomic control in patients with Parkinson's disease: a randomized clinical trial protocol	2021	Chile	Trial	Traditional	Not reported
10	Marques NR et al. [9]	Effects of automatic mechanical peripheral stimulation on gait biomechanics in older adults with Parkinson's disease: a randomized crossover clinical trial	2022	Brasil	Trial	Traditional	Not reported

### 1.1 This scoping review aimed to

This scoping review aimed to comprehensively map and synthesize the existing literature on non-pharmacological interventions for managing gait abnormalities in Parkinson's disease, with a focus on exploring the range of interventions, their effectiveness, and the gaps in current research.

## 2 Methods

The present scoping review was conducted following the JBI methodology [15]for scoping reviews. The Preferred Reporting Items for Systematic reviews and Meta-Analyses extension for Scoping Reviews (PRISMA-ScR) [16] Checklist for reporting was used.

### 2.1 Research team

To support robust and clinically relevant results, the research team included authors with expertise in evidence synthesis, quantitative and qualitative research methodology, sport and musculoskeletal rehabilitation.

### 2.2 Review question

We formulated the following research question: “ We formulated the following research question: What is the effectiveness of non-pharmacological interventions in managing gait abnormalities in Parkinson's disease, and what are the common types of interventions studied in the existing literature?”.

### 2.3 Eligibility criteria

Studies were eligible for inclusion if they met the following Population, Concept, and Context (PCC) criteria.

*Population.* Participants diagnosed with Parkinson's disease.

*Concept.* Non-pharmacological interventions targeting gait abnormalities, including but not limited to exercise, physical therapy, virtual reality, robotics, and automated mechanical peripheral stimulation (AMPS).

*Context.* Studies conducted in any clinical or research setting that evaluated the effectiveness of the interventions on gait outcomes in individuals with Parkinson's disease.

### 2.4 Exclusion criteria

Studies that did not meet the specific PCC criteria were excluded.

### 2.5 Search strategy

An initial limited search of MEDLINE was performed through the PubMed interface to identify articles on the topic and then the index terms used to describe the articles were used to develop a comprehensive search strategy for MEDLINE. The search strategy, which included all identified keywords and index terms, was adapted for use in Cochrane Central, Scopus, PEDro. In addition, grey literature (e.g. Google Scholar, direct contacts with experts in the field) and reference lists of all relevant studies were also searched. Searches were conducted on 23 June 2023 with no date limitation.

### 2.6 Study selection

Once the search strategy has been completed, search results were collated and imported to EndNote V.X9 (Clarivate Analytics). Duplicates were removed using the EndNote deduplicator before the file containing a set of unique records is made available to reviewers for further processing. The selection process consisted of two levels of screening using Rayyan QCRI online software12: [1] a title and abstract screening and [2] a full-text selection. For both levels, two authors independently screened the articles with conflicts resolved by a third author.

The entire selection process and reasons for the exclusion were recorded and reported according to the latest published version of the Preferred Reporting Items for Systematic Reviews and Meta-analyses (PRISMA 2020) flow diagram.

### 2.7 Data extraction and data synthesis

Data extraction was conducted using an ad-hoc data extraction form which was developed a priori, based on the JBI data extraction tool. Key information (authors, country, year of publication, study design, patients characteristics, PFD, type of intervention and related procedures) on the selected articles were collected. Descriptive analyses were performed, and the results were presented in one ways:

Numerically. Studies identified and included were reported as frequency and percentage, and the description of the search decision process was mapped. In addition, extracted data were summarized in tabular and diagrammatic form according to the main characteristics (see Table 2).

**Table 2:** Types of interventions.

Study	Participants	Intervention	Outcomes
Kleiner A et al., 2015	35 subjects with PD35 healthy subjects (control)	Automated Mechanical Peripheral Stimulation (AMPS)	- Variation of spatiotemporal gait parameters (pre and post-AMPS)- Correlation between clinical status of PD patients (H&Y) and percentage of improvement in gait parameters after AMPS.
Stocchi F et al., 2015	18 patients with PD15 age-matched healthy individuals (control group)	Automated Mechanical Peripheral Stimulation (AMPS)	- Improved walking velocity - Positive effect on step and stride length - Increased walking stability, measured by stride length - Positive changes in clinical scales
Pagnussat AS et al., 2018	33 subjects with Parkinson's Disease (PD) 16 subjects in AMPS group 17 subjects in AMPS SHAM group 14 healthy age-matched reference subjects	Automated Mechanical Peripheral Stimulation (AMPS)	- Increased Brain-Derived Neurotrophic Factor (BDNF) serum levels - Decreased Cortisol serum levels - Improved gait velocity - Increased stride length - Improved Timed Up and Go (TUG) performance
Galli M et al., 2018	14 patients with Parkinson's Disease (PD) - AMPS group 14 patients with Parkinson's Disease (PD) - AMPS SHAM group 32 healthy subjects - Control Group	Automated Mechanical Peripheral Stimulation (AMPS)Placebo AMPS (SHAM)	- Significant improvements in gait variables, including spatio-temporal and kinematic parameters, after the first AMPS session and again after the sixth session. - Changes in the shuffling steps pattern, increasing the range of motion (ROM) of hip, knee, and ankle joints during the gait cycle.
Kleiner AFR et al., 2018	30 subjects- AMPS group (n = 15)- AMPS sham group (n = 15)	Automated Mechanical Peripheral Stimulation (AMPS) and AMPS sham	- AMPS decreased gait variability in PD and FOG subjects during single and dual tasks.
Prusch JS et al., 2018	33 subjects with Parkinson's Disease (PD)	Automated Mechanical Peripheral Stimulation (AMPS) or AMPS SHAM (placebo)	- No significant improvement in center of pressure parameters related to postural control with AMPS treatment in individuals with PD and freezing of gait (FOG).
Pinto C et al., 2018	30 subjects with Parkinson's Disease (PD) 15 subjects in AMPS group 15 subjects in AMPS SHAM group 14 healthy age-matched reference subjects	Automated Mechanical Peripheral Stimulation (AMPS) or AMPS SHAM (placebo)	- No significant differences in spatiotemporal gait parameters and lower limb range of motion between AMPS and AMPS SHAM groups. - AMPS group showed significant improvements in spatiotemporal gait parameters and hip rotation range of motion. - AMPS SHAM group did not show improvement
Pagnussat AS et al., 2020	25 participants with Parkinson's Disease (PD) and Freezing of Gait (FOG)	Automated Mechanical Peripheral Stimulation therapy (AMPS)	- No significant changes in brain activity during task-based functional MRI (fMRI) after AMPS treatment.- Increased resting-state functional connectivity between basal ganglia and sensory-related brain areas (insular and somatosensory cortices) after real AMPS. - Improved gait velocity after real AMPS treatment. - Positive correlation between gait velocity and increased connectivity between sensory, motor, and supplementary motor cortices.
Zelada-Astudillo N et al., 2021	Older volunteers with Parkinson's Disease (PD)	12-week program of: - Physical exercise alone (Exercise group) - Combination of Physical exercise and Automated Peripheral Mechanical Stimulation (AMPS) (AMPS + Exercise group)	- Comparison of aerobic capacity, cardiac autonomic control, and gait parameters between the two groups. - Assessing improvements in quality of life for both groups. - Expectation of greater improvements in the AMPS + Exercise group.
Marques NR et al., 2022	28 subjects with Parkinson's Disease (PD)	Automated Mechanical Peripheral Stimulation (AMPS) and Sham intervention	- Increased muscle activation in gastrocnemius lateralis (GL) and tibialis anterior (TA) muscles during walking after AMPS intervention. - Reduced TA activation before and after heel strike and before toe-off after sham intervention. - Shorter time taken to complete the Timed Up and Go (TUG) test after AMPS intervention. - No significant changes in gait kinematics after AMPS intervention.

Legend: AMPS: Automated Mechanical Peripheral Stimulation, BDNF: Brain-Derived Neurotrophic Factor, FOG: Freezing of Gait, GL: Gastrocnemius Lateralis, PD: Parkinson's Disease, ROM: Range of Motion, SHAM: Placebo AMPS, TUG: Timed Up and Go.

## 3 Results

**Fig. 1:**
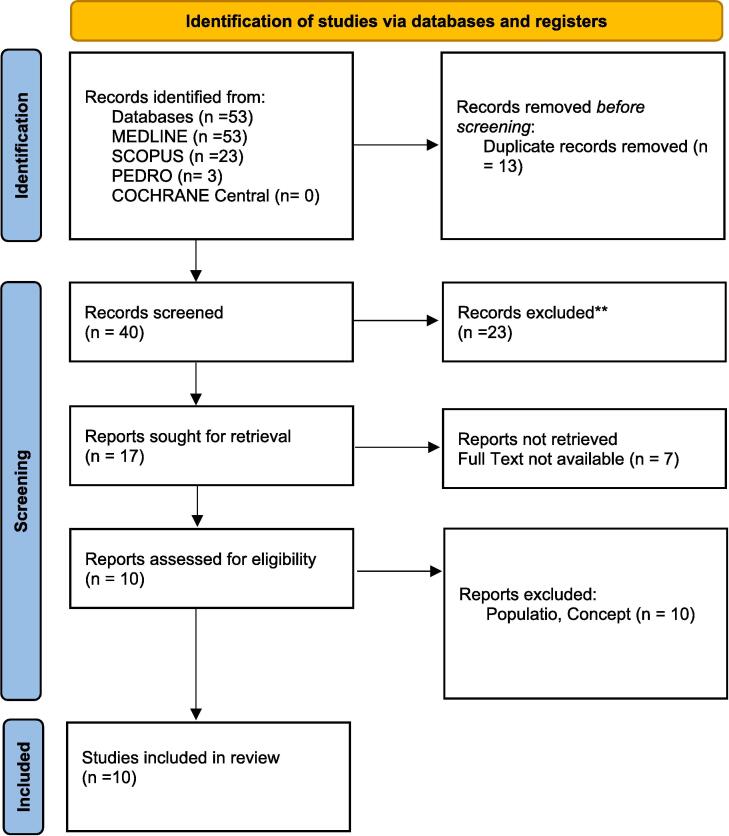
Preferred reporting items for systematic reviews and *meta*-analyses 2020 (PRISMA) flow-diagram.

As presented in the PRISMA 2020-flow diagram (Fig. 1), from 53 records identified by the initial literature searches, 43 were excluded and 10 articles were included.

In this comprehensive scoping review, multiple studies evaluating the effects of Automated Mechanical Peripheral Stimulation (AMPS) on individuals with Parkinson's disease (PD) and freezing of gait (FOG) were examined. The studies explored various aspects of AMPS, ranging from gait variability and spatiotemporal parameters to neurochemical factors and brain activity.

Kleiner et al. (2015) demonstrated that AMPS intervention significantly reduced gait variability in PD and FOG subjects during both single and dual-task conditions, making it an effective add-on therapy for treating gait abnormalities.

Stocchi et al. (2015) found that AMPS led to improved walking velocity, positive effects on step and stride length, and increased walking stability, as measured by stride length, indicating its potential as a promising intervention for PD patients.

Pagnussat et al. (2018) investigated the effects of AMPS on neurochemical factors and gait parameters. They observed increased levels of Brain-Derived Neurotrophic Factor (BDNF), decreased Cortisol levels, and improvements in gait velocity and Timed Up and Go (TUG) performance after AMPS treatment, suggesting positive effects on both gait performance and neurochemical markers.

Galli et al. (2018) revealed significant improvements in gait parameters, including spatiotemporal and kinematic parameters, after the first and sixth AMPS sessions. Additionally, AMPS positively impacted the shuffling steps pattern by increasing the range of motion (ROM) of hip, knee, and ankle joints during the gait cycle.

Prusch et al. (2018) however, did not find significant improvements in postural control after AMPS treatment in PD patients with freezing of gait (FOG), indicating that AMPS may not have a significant positive effect on postural control in these individuals.

Pinto et al. (2018) compared AMPS and AMPS sham interventions and reported no significant differences in spatiotemporal gait parameters and lower limb range of motion between the two groups. However, the AMPS group demonstrated significant improvements in spatiotemporal gait parameters and hip rotation range of motion, while the AMPS sham group did not show improvement.

Pagnussat et al. (2020) investigated the long-term effects of AMPS on brain activity and connectivity. Although AMPS did not significantly change brain activity, it increased resting-state functional connectivity between basal ganglia and sensory-related brain areas. AMPS treatment also improved gait velocity, with a positive correlation between gait velocity and increased connectivity between sensory, motor, and supplementary motor cortices.

Zelada-Astudillo et al. (2021) compared the effects of a 12-week program of physical exercise alone and a combination of physical exercise and AMPS on various parameters in PD patients. Detailed results were not provided in the summary.

Marques et al. (2022) demonstrated increased muscle activation in gastrocnemius lateralis (GL) and tibialis anterior (TA) muscles during walking after AMPS intervention. Additionally, there was a reduction in TA activation after sham intervention, and the Timed Up and Go (TUG) test time was significantly shorter after AMPS intervention.

Overall, these studies suggest that AMPS shows promise as an effective intervention for improving gait abnormalities and functional performance in individuals with Parkinson's disease. Further research is needed to explore its potential in various aspects of PD rehabilitation.

## 4 Discussion

The findings from the scoping review of studies investigating the effects of Automated Mechanical Peripheral Stimulation (AMPS) on individuals with Parkinson's disease (PD) and freezing of gait (FOG) provide valuable insights into the potential benefits and limitations of this therapeutic approach. In this discussion, we will critically analyze the results and address the implications of AMPS as a rehabilitative intervention for PD patients. Firstly, the results of Kleiner A et al.,2015 suggest that AMPS is effective in reducing gait variability in PD and FOG subjects during both single and dual-task conditions. This finding is noteworthy as freezing of gait is a challenging symptom to address, and the observed improvements in gait variability may contribute to enhanced mobility and reduced fall risk in these patients. However, it is essential to consider the sample size and potential confounding factors that may influence the outcomes. The study by Stocchi et al. (2015) highlights the positive effects of AMPS on walking velocity, step and stride length, and walking stability in PD patients. These improvements in gait parameters have significant implications for the overall quality of life of individuals with PD, as gait impairments often lead to reduced independence and increased disability. The observed positive changes in clinical scales further support the potential clinical relevance of AMPS in PD management. On the other hand, Prusch et al. (2018) reported no significant improvement in postural control with AMPS treatment in individuals with PD and FOG. This finding raises questions about the effectiveness of AMPS in addressing postural stability, which is critical for maintaining balance and preventing falls in PD patients. Further investigation is necessary to determine the factors contributing to these results and explore alternative strategies for improving postural control in this population. The study by Pagnussat et al. (2018) indicates that AMPS treatment leads to increased Brain-Derived Neurotrophic Factor (BDNF) levels and decreased Cortisol levels, along with improvements in gait velocity and Timed Up and Go (TUG) performance. These neurochemical and gait-related changes suggest that AMPS may exert beneficial effects on neural plasticity and functional mobility in PD patients. However, the underlying mechanisms responsible for these changes warrant further investigation. Moreover, the results from Pagnussat AS et al.'s., 2018 study also demonstrated increased resting-state functional connectivity between basal ganglia and sensory-related brain areas after AMPS treatment. This finding provides valuable insights into the potential neural adaptations induced by AMPS and its influence on sensorimotor integration. Nevertheless, it remains essential to elucidate the specific neural pathways involved and their functional implications. The study by Pinto C et al. comparing AMPS and AMPS sham interventions revealed significant improvements in spatiotemporal gait parameters and hip rotation range of motion in the AMPS group. These findings suggest that AMPS may have a specific effect on gait biomechanics and joint mobility. However, the lack of significant differences between AMPS and AMPS sham groups in other gait parameters raises questions about the specificity of AMPS as an intervention and the potential placebo effects associated with sham interventions. Additionally, Zelada-Astudillo et al.'s. (2021) study comparing a combination of physical exercise and AMPS with exercise alone emphasizes the need for exploring the synergistic effects of AMPS when combined with other rehabilitation strategies. However, the absence of detailed results in the summary limits our ability to draw definitive conclusions about the potential benefits of the combined intervention. Lastly, the study by Marques NR et al. (2022) provides valuable insights into the impact of AMPS on muscle activation and gait performance. The observed increase in muscle activation during walking and the shorter Timed Up and Go (TUG) test time after AMPS intervention suggest functional improvements in lower limb control and mobility. However, the lack of significant changes in gait kinematics raises questions about the specific biomechanical changes induced by AMPS. In conclusion, the scoping review of studies examining the effects of AMPS on PD patients and freezing of gait reveals promising results in terms of gait improvements, neurochemical changes, and functional outcomes. However, several limitations, such as small sample sizes and the lack of consistent findings in some studies, warrant further research to validate and better understand the therapeutic potential of AMPS in PD rehabilitation [21]. The identified gaps in the literature call for larger-scale randomized controlled trials with standardized protocols to clarify the specific mechanisms underlying AMPS effects and to optimize its integration into comprehensive PD management strategies.

### 4.1 Research implications and suggestions for clinical practice

The scoping review highlights the potential benefits of Automated Mechanical Peripheral Stimulation (AMPS) in Parkinson's disease (PD) and freezing of gait (FOG). Further research is needed to understand the underlying mechanisms, assess long-term effects, and compare AMPS with other therapies. Standardizing AMPS protocols and considering patient stratification can enhance clinical practice. Integrating AMPS into rehabilitation programs may improve gait and functional outcomes for PD patients, but ongoing research is essential to establish its definitive role in clinical practice.

## 5 Strengths and limitations

Strengths:

1. The scoping review includes a comprehensive examination of various studies on the effects of Automated Mechanical Peripheral Stimulation (AMPS) in Parkinson's disease (PD) and freezing of gait (FOG), providing a broad overview of the existing literature.
2. The review encompasses a diverse range of outcomes, including gait parameters, neurochemical markers, brain activity, and functional performance, allowing for a comprehensive understanding of AMPS' potential impact on PD patients.
3. The studies included in the review employ different study designs, including randomized controlled trials and longitudinal studies, enhancing the reliability and validity of the findings.
4. The review provides important insights into the potential role of AMPS as an add-on therapy or complementary approach to improve gait abnormalities in PD patients.

Limitations:

1. Some studies included in the review may have small sample sizes, limiting the generalizability of the findings to larger populations of PD patients.
2. Variations in AMPS protocols and interventions across studies may influence the consistency and comparability of the results.
3. The review may be subject to publication bias, as studies with positive results are more likely to be published than those with negative or null findings.
4. The scoping review may not have accounted for studies published after the literature search was conducted, potentially missing out on more recent relevant studies.
5. The quality of individual studies may vary, which could impact the overall strength of evidence and the validity of the conclusions drawn from the review.

In summary, the scoping review provides valuable insights into the potential benefits of AMPS in PD and FOG. However, caution should be exercised in interpreting the findings due to the limitations identified, and further high-quality research is warranted to establish the effectiveness and clinical implications of AMPS in the management of PD patients.

Answering evidence gap:

• Further research is needed to understand the optimal AMPS protocol for specific PD subtypes and disease stages.
• Mechanistic studies are required to elucidate the neuroplastic changes induced by AMPS and its impact on brain networks.
• Larger and well-controlled trials are essential to confirm the effectiveness and safety of AMPS in a broader PD population.
• Comparative studies with other rehabilitation approaches can determine the unique advantages of AMPS in gait improvement.
• Real-world implementation studies are necessary to evaluate the feasibility and practicality of integrating AMPS into routine clinical practice.

### 5.1 Methodology

An extensive search strategy in the main databases with very broad inclusion criteria was conducted. Moreover, to conduct the review we followed the JBI manual, to describe the selection process we applied the updated PRISMA 2020, and for reporting we used the PRISMA for Scoping Reviews Checklist.

### 5.2 Clinical practice

In clinical practice, the use of Automated Mechanical Peripheral Stimulation (AMPS) can be considered as an adjunctive therapy for individuals with Parkinson's disease (PD) experiencing gait impairments and freezing of gait (FOG). The strengths of AMPS, based on existing research, suggest that it can offer several benefits for PD patients:

1. Gait Improvement: AMPS has shown to improve gait parameters, including walking velocity, stride length, and walking stability. Implementing AMPS sessions can help enhance gait patterns, leading to increased mobility and reduced risk of falls.
2. Neurochemical Effects: Studies have demonstrated that AMPS can increase Brain-Derived Neurotrophic Factor (BDNF) levels and decrease Cortisol levels in PD patients. These neurochemical changes may contribute to neuroplasticity and potentially support neural repair processes.
3. Non-Invasive and Safe: AMPS is a non-invasive intervention that does not require medications or surgery, making it a safe and well-tolerated treatment option for PD patients.

However, there are some limitations and evidence gaps that need to be considered in clinical practice:

1. Limited Generalizability: Some studies have small sample sizes and diverse AMPS protocols, which may limit the generalizability of the findings. Clinicians should interpret the results cautiously and consider individual patient characteristics.
2. Mechanisms Unclear: The precise neurophysiological mechanisms by which AMPS improves gait and motor function are not fully understood. Further research is needed to elucidate these mechanisms and optimize AMPS application.
3. Long-Term Effects: While short-term improvements have been observed, the long-term sustainability of AMPS benefits requires further investigation. Clinicians should carefully monitor patients' progress over time.
4. Efficacy Comparisons: Comparative studies with other rehabilitation approaches are necessary to determine the specific advantages of AMPS over standard treatments or placebo interventions.
5. Implementation Challenges: Integrating AMPS into routine clinical practice may require specialized training and access to appropriate medical devices. Clinicians should be prepared to address logistical and feasibility challenges.

In conclusion, AMPS shows promise as a complementary therapy for gait improvement in PD patients. However, its integration into clinical practice should be informed by the existing evidence, and further research is needed to establish its long-term efficacy, safety, and optimal application. Clinicians should consider individual patient needs and preferences when incorporating AMPS into the overall treatment plan for PD and closely monitor the outcomes to provide the best possible care.

## 6 Conclusions

AMPS holds promise as an effective adjunctive therapy for improving gait parameters in Parkinson's disease (PD) patients, particularly those experiencing freezing of gait. It has demonstrated positive effects on gait velocity, stride length, and walking stability, leading to enhanced mobility and functional performance. However, further research is needed to establish its long-term efficacy and elucidate the underlying neurophysiological mechanisms.

## Declaration of Competing Interest

The authors declare that they have no known competing financial interests or personal relationships that could have appeared to influence the work reported in this paper.
